# The Role of High Concentrations of Homocysteine for the Development of Fetal Growth Restriction

**DOI:** 10.1055/s-0042-1743093

**Published:** 2022-02-17

**Authors:** Andrey Gaiday, Lazzat Balash, Akylbek Tussupkaliyev

**Affiliations:** 1 West Kazakhstan Marat Ospanov Medical University, Aktobe, Kazakhstan; 2 L.N.Gumilyov Eurasian National University, Nur-Sultan, Kazakhstan

**Keywords:** fetal growth restriction, homocysteine, hyperhomocysteinemia, prediction of fetal growth restriction, restrição de crescimento fetal, homocisteína, hiperhomocisteinemia, predição de restrição de crescimento fetal

## Abstract

**Objective** To assess homocysteine (Hcy) levels in the three trimesters of pregnancy in women with fetal growth restriction (FGR) and to evaluate the role of Hcy as a possible predictor of FGR.

**Methods** A total of 315 singleton pregnant women were included in the present prospective cohort study and were monitored since the 1 ^st^ trimester of pregnancy before delivery. Newborns were monitored for the first 7 days of life. Patients who had risk factors for FGR were excluded. Fetal growth restriction was defined according to uterine fundal height (< 10 percentile), ultrasound fetometry (< 5 percentile), and anthropometry of newborns (< 5 percentile). The concentrations of Hcy were detected at between 10 and 14, between 20 and 24, and between 30 and 34 weeks of pregnancy by enzyme-linked immunosorbent assay (ELISA). Receiver operating characteristics (ROC) curve test and diagnostic odds ratio (DOR) were performed to evaluate the results of ELISA.

**Results** The concentration of Hcy in patients with FGR was 19.65 umol/L at between 10 and 14 weeks, compared with 9.28 umol/L in patients with normal fetal growth ( *p* < 0.0001). The optimal cut-off level for Hcy in the 1 ^st^ trimester of pregnancy was > 13.9 umol/L with AUC 0.788, sensitivity of 75%, specificity of 83.6%, and DOR of 15.2.

**Conclusion** Assessment of serum Hcy concentration may be used as a predictor of FGR, with the highest diagnostic utility in the 1 ^st^ trimester of pregnancy.

## Resumo

**Objetivo** Avaliar os níveis de homocisteína (Hcy) em três trimestres da gravidez em mulheres com restrição de crescimento fetal (FGR, na sigla em inglês) e avaliar o papel da Hcy como possível preditor de FGR.

**Métodos** Um total de 315 gestantes solteiras foram incluídas no presente estudo de coorte prospectivo e monitoradas desde o 1° trimestre de gravidez antes do parto. Os recém-nascidos foram acompanhados durante os primeiros 7 dias de vida. Pacientes que apresentam fatores de risco para FGR foram excluídos. A FGR foi definida de acordo com a altura do fundo do útero (< percentil 10), ultrassonografia fetometria (< percentil 5) e antropometria dos recém-nascidos (< percentil 5). As concentrações de Hcy foram detectadas entre 10 e 14, entre 20 e 24 e entre 30 e 34 semanas de gravidez por ensaio de imunoabsorção enzimática (ELISA, na sigla em inglês). O teste da curva das características de operação do receptor (ROC, na sigla em inglês) e a razão de chances de diagnóstico (DOR, na sigla em inglês) foram realizados para avaliar os resultados do ELISA.

**Resultados** A concentração de Hcy em pacientes com FGR foi de 19,65 umol/L entre 10 e 14 semanas, em comparação com 9,28 umol/L em pacientes com crescimento fetal normal ( *p* < 0,0001). O nível de corte ideal para Hcy no 1° trimestre da gravidez foi > 13,9 umol/L com AUC 0,788, sensibilidade de 75%, especificidade de 83,6%, e DOR 15,2.

**Conclusão** A avaliação da concentração sérica de Hcy pode ser usada como um preditor de FGR, com maior utilidade diagnóstica no 1° trimestre de gravidez.

## Introduction

Fetal growth restriction (FGR) occurs when the fetus does not reach its intrauterine potential for growth and development as a result of compromise in placental function. 1 According to various sources, the incidence of FGR is between 5 and 10% worldwide, and is the second cause of perinatal mortality. 1 The risk of death of newborns with FGR increases between 2 and 4 times, and the negative outcomes of childbirth are manifested in newborns as hypothermia, hypoglycemia, hyperglycemia, persistent pulmonary hypertension, pulmonary hemorrhage, polycythemia, stillbirth, and intranatal asphyxia. 2

In the process of growth, the fetus produces hemodynamic and metabolic changes, in which an adequate trophoblast invasion is an important component, 3 and endothelial and subendothelial changes can contribute to their violation. 4 Today, it is known that the underlying causes of FGR are genetic, placental, fetal, and maternal factors. 2 5 6

An important physiological process that ensures normal perfusion of the placenta is the invasion of trophoblast villi and the reshuffle of the cytotrophoblast from the epithelial to the endothelial phenotype, which is called pseudovasculogenesis. 7 Subsequently, the remodeling of the spiral arteries occurs, while the cytotrophoblast increases the expression of vascular endothelial growth factor (VEGF) and placental growth factor (PLGF). 8 Nowadays, serum biomarkers are increasingly preferred for the prediction and diagnosis of FGR, of which the most commonly used are pregnancy-associated plasma protein-A (PAPP-A), α-fetoprotein (AFP), placental growth factor (PLGF), and soluble fms-like tyrosine kinase-1 (sFlt-1). 6 9 10 11 12 13 Along with well-known biomarkers, in the last decade, studies indicated the possibility of using serum homocysteine (Hcy) for the prediction and diagnosis of preeclampsia (PE) and FGR; 14 15 16 17 18 however, there are no studies available from the Kazakhstan population. The development of FGR in hyperhomocysteinemia (HHcy) may be due to the elevation of asymmetric dimethylarginine (ADMA) levels since Hcy has an inhibitory effect on ADMA metabolism. Injury to endothelial cells is also associated with HHcy, which leads to changes in the coagulation system, platelet activation, and thrombogenesis. 19

In this study, we hypostatized that HHcy could be considered as an additional marker for the prediction and diagnosis of FGR. The aim of our study was to assess Hcy levels in the 3 trimesters (10–14, 20–24, and 30–34 weeks) of pregnancy in women with FGR and to evaluate the role of Hcy as a possible predictor of FGR. Confirmation of the hypothesis can be used to identify groups of patients with HHcy, as well as to search for the prevention of FGR from the 1 ^st^ trimester of pregnancy.

## Methods

The present study was part of the scientific program “Development and scientific substantiation of new technologies for protecting the health of newborns” and was approved by the Local Ethical Committee (protocol no. 12 28/12/2015) and registered at the National Center of Science and Technology Evaluation of the Republic of Kazakhstan (0107RKI00477). All patients signed a written informed consent to participate in the study.

All patients were invited to participate in this prospective cohort study at the Aktobe city outpatient department during antenatal and postpartum periods and in the regional perinatal center (Aktobe, Kazakhstan) during the intrapartum and postpartum periods between April 2016 and February 2018. Of the 615 subjects, consent to participate in the study was obtained from 360 patients who were included in the study. A total of 45 patients did not complete the study: 7 (1.9%) had miscarriages; 6 (1.7%) changed their place of residence and were not available for observation; 8 (2.2%) refused to continue the study; and, subsequently, 24 (6.7%) had hypertensive disorders or diabetes mellitus and were excluded according to the study protocol. Finally, we studied 315 pregnancies from the 1 ^st^ trimester (between 10and 14 weeks) before birth. Newborns were monitored for the first 7 days of life. The inclusion criteria were age between18 and 40 years old, singleton pregnancy, normal fetal anatomy, body mass index (BMI) between 19 and 30 kg/m ^2^ , without preeclampsia. We excluded patients, who had confounders for FGR such as multiple pregnancies, fetuses with chromosomal anomalies, FGR in a previous pregnancy, diabetes, hematologic and autoimmune diseases, congenital disorders, lung diseases, kidney failure, history of chronic hypertension or preeclampsia, smoking, alcohol or drug abuse, and low socioeconomic status. 1 2 5 20 21

The gestational age was determined by the date of the last menstruation by the Naegele rule, and by using a Samsung Medison RS80-A ultrasound machine (Samsung Medison, South Korea), transvaginal and transabdominal ultrasound fetometry were used, which determined the crown-rump length (CRL) and were compared with known values. 22 23 In case of difference between the gestational age (according to the date of the last menstruation) and ultrasound fetometry > 5 days, the gestational age was determined according to ultrasound data. 24 25 Serial examination of the measurements of uterine fundal height and transabdominal ultrasound fetometry (abdominal circumference, head circumference, and femur length) every 4 weeks from 20 weeks of gestation were performed. Fetal growth restriction was determined as a primary < 10 percentile or fetal growth arrest at initial normal rates of uterine fundal height in gravidogram 26 and/or < 5 percentile by the standard curve by ultrasound fetometry, 27 which were necessarily confirmed by < 5 percentile by the standard curve of the body weight, height, and BMI of the newborns regarding gestational age. 28 The diagnosis of FGR was rejected if the anthropometric parameters of newborns were > 5 percentile by the standard curve and the data were not evaluated in the study. The conditions of newborns were assessed using the Apgar scale and a complete clinical examination was performed.

Homocysteine concentrations were determined at between 10 and 14, between 20 and 24, and between 30 and 34 weeks of pregnancy by enzyme-linked immunosorbent assay (ELISA). Venous blood samples (5 ml) were collected after overnight fasting and cancellation of folic acid supplements, drugs, or dietary supplements containing S–adenosyl-L-methionine intake for 14 days, into an AVATUBE vacuum container with an activator gel (Eco Pharm International, Kazakhstan), then the samples were centrifuged at 1,500 rpm no later than 30 minutes after the sample collection to split the pallets. The samples were stored at – 20°C for up to 8 weeks. Monoclonal antibodies Homocysteine EIA microtiter plate with ELISA reagents were used to detect Hcy (Axis-Shield Diagnostics Ltd, United Kingdom). The optical density was measured by photometry at 450 nm using a Dialab ELX808IU microplate reader (Dialab, Austria). Concentrations of the Hcy amino acid were obtained from the optical density data by applying a method of the standard curve. 29

Statistical analyses were performed by using Statistica 12.0 software (Stat Soft Inc., USA). The type of distribution was evaluated by the Shapiro-Wilk test. For data with an abnormal distribution, median (Me) with 25 to 75 interquartile range (IQR) were determined. Analyses for independent two variables were performed with the Mann-Whitney U-test and the Fisher exact test (two-tailed), whereas for the dependent variables the Friedman and Tukey post-hoc tests were applied. To determine the optimal cut-off levels for the concentration of Hcy, a receiver operating characteristics (ROC) curve test was applied, performed by the statistical processing program Med Calc 19.1 (Med Calc Software Ltd). Receiver operating characteristics analyses included evaluation of the area under the curve (AUC), sensitivity (Se), specificity (Sp), Youden index (J), negative likelihood ratio (-LR), and positive likelihood ratio (+LR). The diagnostic odds ratio (DOR) was calculated and evaluated for study groups according to a previously published protocol. 30 Receiver operating characteristics curves have been compared by the DeLong et al. test. 31

The level of statistical significance was defined as *p* < 0.05.

The present study was approved by the Local Ethical Committee protocol no. 12 of December 28, 2105, at the West Kazakhstan Marat Ospanov Medical University. The present study was conducted in accordance with the Helsinki Declaration.

## Results

After the clinical manifestation of FGR, the patients were divided into 2 groups: the FGR group, with 12 (3.8%) cases, and the control group, with 305 (96.2%) cases. There were no differences between the study groups in anamnestic, clinical, and ethnic data ( Table 1 ).

**Table 1:** Clinical characteristics of the patients in the FGR and Control groups

	FGR group ( *n* = 12)	Control group ( *n* = 305)	*p-value*
Age, years old, median (IQR)	27 (25–32)	28 (25–31)	0.883 a
Menarche, years old, median (IQR)	14 (13–14)	13 (13–14)	0.587 a
Abnormal menstrual function, *n*	−	6 (1.9%)	−
Nulliparous, *n*	3 (25%)	65 (21.3%)	0.999 b
Multiparous, *n*	9 (75%)	240 (78.7%)	0.999 b
Previous abortions, *n*	4 (33.3%)	70 (22.9%)	0.484 b
Previous miscarriages, *n*	3 (25%)	71 (23.3%)	1.0 b
Preterm labor, *n*	2 (16.6%)	16 (5.2%)	0.143 b
BMI, кg/m ^2^ , median (IQR)	21.4 (19.7–22.9)	22.4 (20.6–24.6)	0.219 a
Gestational age at admission, weeks, median (IQR)	12.5 (10–13.5)	12 (11–13)	0.832 a
Gestational age at delivery, weeks, median (IQR)	39 (37.5–40)	39 (38–40)	0.69 a

Abbreviations: BMI, body mass index; FGR, fetal growth restriction; IQR, interquartile range.

a Mann-Whitney U-test

b two-tailed Fisher test

The newborns had a lower median of weight, height, BMI, and Apgar score, and were more often transferred to the intensive care unit in the FGR group ( *p* < 0.05) ( Table 2 ). There were no differences in stillbirth, neonatal death, and malformations between FGR and the control group (p > 0.05) ( Table 2 ).

**Table 2:** Clinical characteristics newborns in the FGR and Control groups

	FGR group ( *n* = 12)	Control group ( *n* = 305)	*p-value*
Weight, grams, median (IQR)	2200 (2160–2430)	3420 (3130–3730)	< 0.0001 a
Height, centimeters, median (IQR)	47.5 (46.5–50)	53 (52–55)	< 0.0001 a
BMI, kg/m ^2^ , median (IQR)	9.57 (8.8–10.77)	12 (11.46–12.57)	< 0.0001 a
Apgar score, 1 ^st^ minute, median (IQR)	7.5 (5.5–9)	9 (8–9)	< 0.0001 a
Apgar score, 5 ^th^ minute, median (IQR)	8.5 (7.5–10)	10 (9–10)	0.002 a
Hospitalization in the intensive care unit, *n*	4 (33.3%)	14 (4.6%)	0.002 b
Stillbirth, *n*	1 (8.3%)	1 (0.3%)	0.074 b
Neonatal death, *n*	1 (8.3%)	1 (0.3%)	0.074 b
Malformations, *n*	1 (8.3%)	2 (0.6%)	0.109 b

Abbreviations: BMI, body mass index; FGR, fetal growth restriction; IQR, interquartile range.

a U-test Mann-Whitney

b two-tailed Fisher test

The medians of Hcy concentration in the patients with FGR were 19.65 umol/L at between 10–14 weeks, 18.49 umol/L at between 20 and 24 weeks, and 15.36 umol/L at between 30 and 34 weeks, which is significantly higher when compared with the control group ( *p* < 0.05) ( Table 3 ). In addition, we found that Hcy concentration was similar during the entire gestation within the FGR group, indicating abnormal stability, compared with decreasing concentrations of Hcy during gestation in the control group ( Table 3 ).

**Table 3:** Analyses of the serum homocysteine concentrations during pregnancy in the FGR and Control groups

	Hcy concentration in 10–14 weeks Median (IQR) (umol/L)	Hcy concentration in 20–24 weeks Median (IQR) (umol/L)	Hcy concentration in 30–34 weeks Median (IQR) (umol/L)	*p-value*
FGR group ( *n* = 12)	19.65 (10.88–22.28)	18.49 (6.39–25.8)	15.36 (7.83–24.87)	0.558 b
Control group ( *n* = 305)	9.28 c (5.17–12.4)	8.21 c (4.12–10.94)	6.83 c (2.8–9.23)	< 0.0001 b
*p-value*	< 0.001 a	< 0.012 a	< 0.001 a	

Abbreviations: FGR, fetal growth restriction; Hcy, homocysteine.

a Mann-Whitney U-test

b Friedman test

c *p* < 0.0001 post-hoc Tukey test (10–14 weeks versus 20–24 weeks versus 30–34 weeks)

**Fig. 1:**
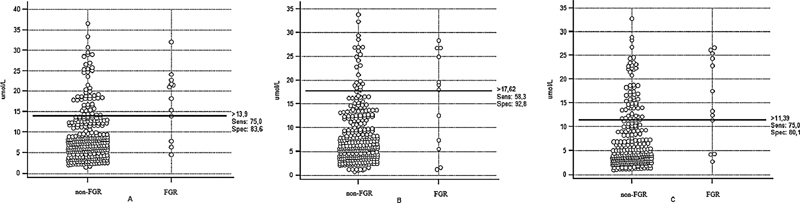
Graphical characteristics of optimal cut-off levels of homocysteine concentrations in the first ( **A** ), second ( **B** ), and third ( **C** ) trimesters of pregnancy.

**Fig. 2:**
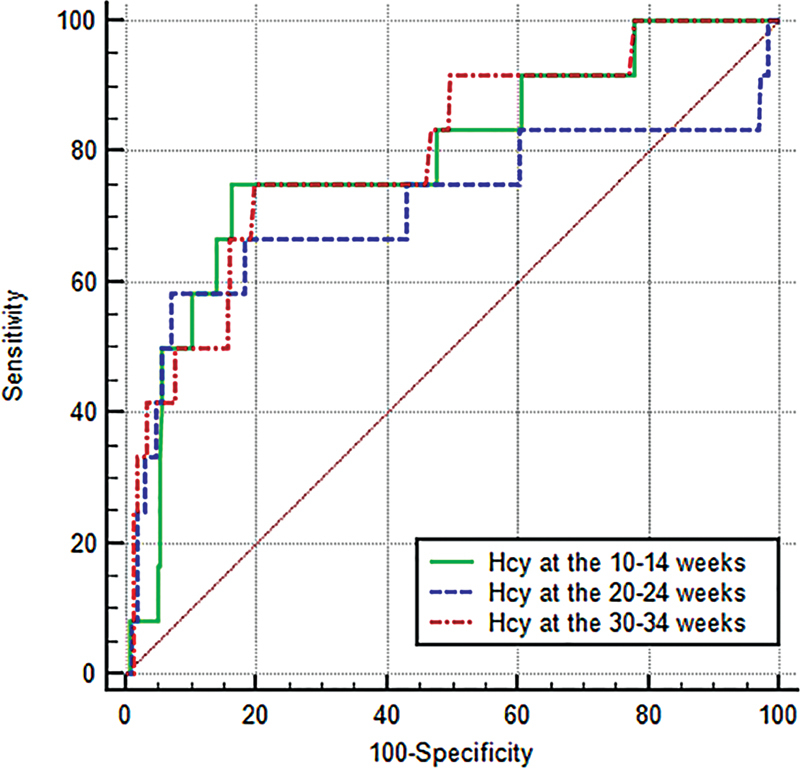
Illustration of comparison of ROC curves for homocysteine (Hcy) concentrations depending on period of gestation.

The results of the analysis of the ROC determined optimal cut-off levels of Hcy concentrations 13.9 umol/L at between 10 and 14 weeks, 17.62 umol/L at between 20 and 24 weeks, and 11.39 umol/L at between 30 and 34 weeks ( Fig. 1 ). There were no significant differences between the AUC depending on the period of gestation ( Fig. 2 ). However, the highest DOR value (17.9) was in Hcy determined at between 20 and 24 weeks of gestation, but with a low sensitivity of 58.3%, compared with Hcy determined at between 10 and 14 weeks, with a DOR of 15.6 with sensitivity of 75% and specificity of 83.6% ( Table 4 ).

**Table 4:** Results of ROC-analysis of homocysteine in different weeks of gestation

Gestational period of Hcy evaluation	AUC	Se (%)	Sp (%)	*p - value*	J - index	+LR (95CI)	-LR (95CI)	DOR
10–14 weeks	0.788 a	75	83.6	< 0.001	0.586	4.58 (3.0–6.9)	0.3 (0.1–0.8)	15.2
20–24 weeks	0.712 a	58.3	92.8	0.049	0.511	8.09 (4.3–15.1)	0.45 (0.2–0.9)	17.9
30–34 weeks	0.799 a	75	80.1	< 0.001	0.551	3.77 (2.5–5.6)	0.31 (0.1–0.8)	12.1

AUC, area under the curve; CI, confidence interval; DOR, diagnostics odds ratio; Hcy, homocysteine; J, Youden's index; -LR, negative likelihood ratio; +LR, positive likelihood ratio; Se, sensitivity; Sp, specificity.

a *p* > 0.05 pairwise comparison of ROC curves.

## Discussion

Results of our cohort study showed that, despite the clinical homogeneity of the groups, 3.8% of the observed patients, without any risk factors, were complicated by FGR. Newborns with FGR had a lower Apgar score and were more often transferred to the intensive care unit, as confirmed by the study by Melchiorre et al., 2 but did not present a higher frequency of stillbirth, malformations, and neonatal mortality.

In our study, the concentrations of serum Hcy in women with FGR were 19.65 umol/L at between 10 and 14 weeks, 18.49 umol/L at between 20 and 24 weeks, and 15.36 umol/L at between 30 and 34 weeks and were significantly different from those of women with normal fetal growth. These results are similar to those of studies by Bergen et al., 32 Vollset et al., 33 who also found significantly higher Hcy concentrations at the 1 ^st^ trimester of pregnancy in women with FGR, and by Furness et al., 34 who investigated high Hcy concentrations at the 2 ^nd^ trimester of pregnancy in women with FGR. Yeter et al., 14 Gadhok et al., 35 and Jiang et al. 17 determined increased Hcy levels in the 3 ^rd^ trimester of pregnancy in women with FGR. However, several previous studies by D'Anna et al., 36 Hogg et al., 37 Cawley et al., 38 and Gomes et al. 39 demonstrated the absence of any difference in serum Hcy levels during pregnancy among women who later developed FGR and those who remained with normal fetal growth.

We also noticed high concentrations of Hcy during pregnancy in the patients who developed FGR, in contrast with pregnancies with normal fetal growth, in which Hcy significantly decreased during pregnancy.

The prognostic and diagnostic role of Hcy for FGR was confirmed by analysis of the ROC, which showed good effectiveness at the 1 ^st^ and 3 ^rd^ trimesters of pregnancy.

Murphy et al. 40 observed that mothers with a Hcy concentration > 8.44 umol/L at 8 weeks of gestation were 3 times more likely to give birth to an infant in the lowest birthweight tertile. In a study by Bergen et al., 32 pregnancy was complicated by FGR at a Hcy concentration > 8.3 umol/L (OR: 1.68 [1.16–2.43]) determined at a gestational age of < 18 weeks. In another study, by Chaudhry et al., 41 Hcy concentrations > 5.0 umol/L were significant for the development of FGR (OR: 1.69 [1.227–2.161]) at 8 weeks of gestational age. The data from previous studies differ significantly from those of our study, in which a significant Hcy concentration in the 1 ^st^ trimester of pregnancy was determined at 13.9 umol/L, and are similar to those of the study by Steegers et al., 42 which indicates that a Hcy concentration > 15 umol/L is significant for the development of FGR.

The role of Hcy assessment in the 2 ^nd^ trimester of pregnancy remains unclear. Maged et al. 16 also suggested a certain role of serum Hcy determination as a prognostic and diagnostic marker for FGR, but in combination with Doppler velocimetry of the uterine artery.

As for the 3 ^rd^ trimester of pregnancy, there were enough case-control studies at the time of the clinical manifestation of FGR, 17 42 43 44 45 but this does not make it possible to predict HFR, since it had already developed.

We have also identified some limitations of the present study. For example, the present study investigated patients with low risk of FGR because the well-known FGR risk factors were added to the exclusion criteria. One of the limitations of the study may also be a relatively small sample size of patients with FGR, but this is a common issue for prospective studies as judged by other publications.

## Conclusion

The results of our study showed that the assessment of serum Hcy concentration at the 1 ^st^ trimesters of pregnancy may be used as a predictor of FGR. Also, we hypothesize that assessment of serum Hcy concentrations in the 2 ^nd^ and 3 ^rd^ trimesters of pregnancy can be an additional marker of FGR.
